# Hand hygiene on gloved hands: is glove integrity compromised by repeated disinfections?

**DOI:** 10.1017/ash.2025.167

**Published:** 2025-05-13

**Authors:** Michelle Doll, Tiffany Zhao, Ian Langford, Barry Rittmann, Patrick R. Ching, Pamela Bailey, Gonzalo Bearman

**Affiliations:** 1 VCU School of Medicine, Richmond, VA, USA; 2 VCU Health System, Richmond, VA, USA; 3 Prisma Health Midlands, Columbia, SC, USA; 4 University of South Carolina School of Medicine Columbia, Columbia, SC, USA

## Introduction

Achieving optimal hand hygiene (HH) while providing high acuity care is difficult given the number of opportunities within a single care episode.^1,2^ Nursing care for patients on contact precautions CP is often bundled, with multiple tasks occurring without performing HH once in personal protective equipment (PPE).^3^ To increase overall HH, some experts have recommended HH on gloved hands during the same patient care episode, acknowledging that removal and replacement of gloves with each opportunity is not feasible in high acuity settings.^4,5^ However, medical exam gloves are Food and Drug Administration approved as single-use only.^6^

Existing data suggests that disinfection of gloves is effective in decreasing glove contamination.^7,8^ However, there are concerns that glove integrity could be compromised after repeated disinfection. Scheithauer et al.^7^ used a water leak test (WLT) to check for leaks after repeat (5×) contamination with *Escherichia coli* and subsequent disinfection, finding that 4/5 brands and 7/100 individual gloves leaked at the end of the manipulations. Shless et al.^8^ also used WLT to evaluate repeated soap and water, alcohol-based hand rub (ABHR), or bleach treatments versus a “control” of untreated gloves from the same brands in the context of PPE shortages. They witnessed wide variability in physical integrity of the gloves tested, and suggested that facilities evaluate glove/disinfectant combinations locally to determine if extended use were safe/appropriate. Garrido-Molina et al.^9^ repeatedly disinfected gloves with seven different types of solutions and subjected gloves to tensile testing, finding (ABHRs) reduced the force required to break gloves. However, the lack of a comparison or dose-response limits interpretability of the data from Scheithauer^7^ and Shless,^8^ and differences in glove effects as measured by tensile testing^9^ are less clinically relevant as gloves are not progressively stretched in clinical care.

We sought to examine glove integrity using WLT to evaluate for clinically meaningful defects in the glove barrier function after repeated disinfections, ranging from five to fifteen, in attempt to demonstrate a dose-response relationship between disinfections and appearance of leaks. We focused on nitrile medical exam gloves and ABHR exclusively as commonly used glove/disinfection combinations in US hospitals.

## Methods

**Figure 1.**
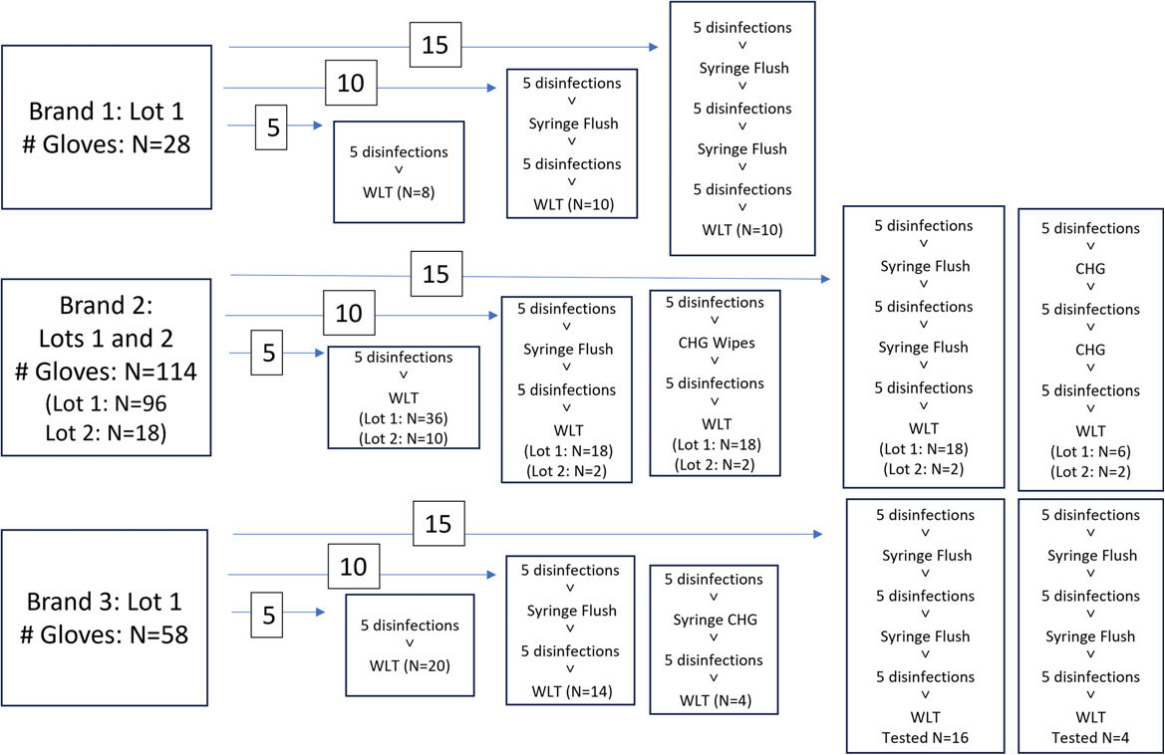
Pre-Treatment Path for Each Glove: Total number of gloves tested by each method are grouped by the number of disinfections and depicted by the numbers inserted over arrows (ie 5, 10, or 15 disinfections). Specific clinical tasks applied to each glove are shown in the 10, and 15 disinfection groups. Total number of gloves tested by each method is in the bottom of each box.

Nitrile medical exam gloves from three different manufacturers currently in use in our facility were used for this study. The gloves tested represented a convenience sample from the primary brand in use (brand 2) in an approximate 2:1 ratio with the other/additional brands (brands 1,3). The gloves were evaluated by two individuals as follows: gloves in the appropriate size were inspected visually for existing defects. Gloves were then donned and disinfected with 62% ethanol AHBR and gently flapped to facilitate air-drying. Between sets of five disinfections (for the 10 and 15 disinfection arms), one of two mock clinical activities was performed: a mock aspiration using a large syringe (without a needle), or a chlorhexidine cleaning activity (gloved hands were wiped with a 2% CHG impregnated cloth) (Figure 1). The tasks were performed to mimic a clinical task that would add friction or chemicals potentially further impacting glove integrity. One of the testers wore a wedding band. One tester was left-handed; the other was right-handed. Both testers wore their usual/appropriately sized gloves and had nails maintained at no more than ¼ inch past the fingertip per our (HH) policy.

After the final planned disinfection, gloves were doffed and immediately affixed to the WLT machine (DipTech Systems Inc., Kent OH) and tested following ASTM standard D5151-19.^10^ Briefly, gloves were attached to mandrel of the machine and secured with a strap, then filled with 1 L of water between 15–30 °C and visually inspected for leaks. The total number and location of leaks were recorded for each glove.

The number of defects were compared against each of the following variables using Fisher’s Exact Test using SAS 9.4 (Cary, NC): glove brand/lot, number of disinfections, clinical activity, glove tester.

## Results

Two hundred individual gloves were tested (100 sets) as shown in Table 1. No glove had more than one leak. No leaks were detected in brand 2 for any number of disinfections/manipulations. There was a significant difference in the quantity of gloves with leaks between glove type and lot (Table 1, P = 0.0016 and *P* = 0.0053 respectively), but not by number of disinfections (*P* = 0.2631).

**Table 1.** Gloves with water leak defects after repeat disinfections by brand/lot

Glove type/Lot	ABHR × 5 leaks/N (%)	ABHR × 10 leaks/N (%)	ABHR × 15 leaks /total (%)	Total leaks /total (%)
Brand 1: Lot 1	0/8 (0%)	0/10 (0%)	1/10 (10%)	1/28 (3.6%)
Brand 2: Lot 1	0/10 (0%)	0/4 (0%)	0/4 (0%)	0/18 (0%)
Brand 2: Lot 2	0/36 (0%)	0/36 (0%)	0/24 (0%)	0/96 (0%)
Brand 3: Lot 1	1/20 (5%)	2/18 (11.1%)	3/20 (15%)	6/58 (10.3%)

N = number individual gloves tested.

In addition, there was no difference in leak occurrence by type of mock clinical task: no task = 1 leak/73 gloves tested, CHG exposure = 2 leaks/24 gloves, syringe flush = 4 leaks/86 gloves (*P* = 0.4077). There was no difference in leak occurrence between right and left hand gloves: 4/96 versus 3/97 respectively, (*P* = 1.0000).

## Discussion

Our WLT test data suggest highly variable glove integrity, that appears to be primarily dependent on glove brand rather than number of disinfections or glove manipulations. The inability to show significant impacts of increasing disinfections or manipulations may be related to the low numbers of gloves in brands 1, 3 and the absence of any leaks in brand 2. Nevertheless, we conclude that repeated disinfection with glove brand 2 is a reasonable practice to improve HH during the in-room care of the same patient.

This study supports the recommendation made by Shless et al.^8^ that individual facilities should evaluate their internal products for repeated disinfection given the highly variable performance of specific glove types. There are multiple reasons why facilities would want to evaluate gloves for repeated usage including contingency plans for supply chain shortages, and repeated disinfection on gloves during the same care encounter (for the same patient) as described here. In addition to improving HH rates during in-room care, performing HH over gloved hands also reduces the amount of waste from repeated doffing and donning of gloves in a single patient encounter. Most facilities do not have access to (WLTs). Rather than each facility evaluating their own products, manufacturers and regulatory bodies should include glove integrity after repeated disinfection in product testing data, and manufacturers should provide evidence-based guidance for appropriate repeated glove use.

This study did not include microbiology data and thus does not support extended glove use *between* patients. Discarding gloves between patients, at the conclusion of each patient care episode, is a standard of care that the infection prevention community strongly supports. The repeated disinfections suggested in this work apply to an individual/same patient in a single care episode, when gloves are not visibly soiled, such as (for example) bundled care for a complex patient on CPs.

This study is limited by the small number of gloves tested across each brand, convenience sampling, and lack of microbiologic data. Existing data suggests disinfection of gloved hands results in greater microbial reductions than disinfection of bare hands.^7^

Despite these limitations, the variability of glove performance and the finding that integrity appears more brand-determined than related to repeated disinfections, are similar to prior studies and may be generalizable. This study provides further support that the benefits of appropriate HH on gloved hands (ie when caring for the same patient in a single care episode), are likely greater than the risks of repeated glove use. Specifically, appropriate HH on gloved hands may allow greater adherence to HH in single patient care episode and decrease in-room transmission between the patient and the immediate environment. More studies are needed to improve the effective and sustainable use of gloves and other types of (PPE) in health care.
